# The Time Profile of Pentraxin 3 in Patients with Acute ST-Elevation Myocardial Infarction and Stable Angina Pectoris Undergoing Percutaneous Coronary Intervention

**DOI:** 10.1155/2014/608414

**Published:** 2014-03-11

**Authors:** Ragnhild Helseth, Svein Solheim, Trine Opstad, Pavel Hoffmann, Harald Arnesen, Ingebjørg Seljeflot

**Affiliations:** ^1^Department of Cardiology, Oslo University Hospital Ullevål, P.O. Box 4956, Nydalen, 0424 Oslo, Norway; ^2^Faculty of Medicine, University of Oslo, P.O. Box 1078, Blindern, 0316 Oslo, Norway; ^3^Section for Interventional Cardiology, Department of Cardiology, Division of Cardiovascular and Pulmonary Diseases, Oslo University Hospital Ullevål, P.O. Box 4956, Nydalen, 0424 Oslo, Norway

## Abstract

*Background*. High levels of Pentraxin 3 (PTX3) are reported in acute myocardial infarction (AMI). *Aim*. To investigate circulating levels and gene expression of PTX3 in patients with AMI and stable angina pectoris (AP) undergoing PCI. *Methods*. Ten patients with AP and 20 patients with AMI were included. Blood samples were drawn before PCI in the AP group and after 3 and 12 hours and days 1, 3, 5, 7, and 14 in both groups. *Results*. Circulating PTX3 levels were higher in AMI compared to AP at 3 and 12 hours (*P* < 0.001 and *P* = 0.003). Within the AMI group, reduction from 3 hours to all later time points was observed (all *P* ≤ 0.001). Within the AP group, increase from baseline to 3 hours (*P* = 0.022), followed by reductions thereafter (all *P* < 0.05), was observed. PTX3 mRNA increased in the AMI group from 3 hours to days 7 and 14 in a relative manner of 62% and 73%, while a relative reduction from baseline to 3 and 12 hours of 29% and 37% was seen in the AP group. *Conclusion*. High circulating PTX3 levels shortly after PCI in AMI indicate that AMI itself influences PTX3 levels. PTX3 mRNA might be in response to fluctuations in circulating levels.

## 1. Introduction

Acute coronary syndrome (ACS) including myocardial infarction with or without ST-elevation (STEMI and NSTEMI) and unstable angina pectoris are major causes of morbidity and mortality worldwide [1]. Rupture of an atherosclerotic coronary plaque and subsequent thrombosis are now accepted as the most frequent underlying pathological process in ACS [2]. Early percutaneous coronary intervention (PCI) with stent implantation and complete revascularization of the affected coronary vessel is currently the treatment of mode to minimize myocardial necrosis and optimize cardiac repair.

ACS is now recognized as the net result of a complex event cascade in which thrombotic and inflammatory processes interact and ultimately lead to an atherosclerotic plaque with subsequent destabilization and rupture [2]. One of the inflammatory markers proven to be of predictive value to both ACS development and established coronary artery disease is the short Pentraxin C-reactive protein (CRP) [2, 3]. In the early 1990s, a new member of the Pentraxin superfamily, the long Pentraxin 3 (PTX3), was identified. Unlike CRP, which is mainly produced by hepatocytes in response to interleukin 6 (IL-6), PTX3 is secreted by a variety of cells like smooth muscle cells, endothelial cells, macrophages, and leukocytes in response to a diversity of proinflammatory signals [4]. Circulating levels of PTX3 have been reported to be elevated in acute myocardial infarction and have further been suggested to be predictive of coronary artery disease (CAD) and CAD mortality; the latter was discussed to be even superior to traditional markers like Troponin T, creatine kinase, and pro-BNP [5–7]. A complete understanding of the actions of PTX3 is not present to date. It appears, however, that PTX3 has both proinflammatory and anti-inflammatory effects, depending on the situation and anatomic location of the action [4]. The immediate secretion of PTX3 during ACS is thought to originate from specific granules of neutrophile granulocytes [4, 8]. It has been reported that the locally secreted PTX3 in the affected coronary artery inhibits P-selectin-dependent leukocyte enrollment and platelet aggregation in an anti-inflammatory manner [4, 8, 9]. Studies on PTX3^−^/^−^ mice have further reported increased atherosclerosis and increased myocardial damage in situations imitating AMI, supporting a cardioprotective theory [10, 11]. On the other hand, PTX3 is known to locally stimulate the classical pathway of the complement cascade, hence theoretically increasing apoptosis of injured cells like ischemic cardiomyocytes [4]. Whether the potential prognostic value of PTX3 in CAD reflects its influence on coagulation and complement activation or if high levels of PTX3 are a protective response reflecting the degree of inflammation and myocardial injury is not fully understood.

The main aim of the present study was to explore and compare the time profile of circulating PTX3 levels to PTX3 mRNA levels in whole blood (circulating leukocytes) in patients with stable angina pectoris (AP) and acute ST-elevation infarction (AMI), all undergoing successful revascularization with PCI and stent implantation. By investigating both AMI and AP patients we wanted to explore a potential effect of the PCI procedure per se on PTX3 release. Relationship between circulating PTX3 levels and the degree of myocardial necrosis and leukocyte count were further assessed.

## 2. Materials and Methods

### 2.1. Subjects and Study Design

Patients between 30 and 75 years, both gender, with AMI (*n* = 20) or stable AP (*n* = 10) admitted to Ullevål University Hospital, Oslo, Norway, were included. All were successfully revascularized achieving normal coronary blood flow during the PCI procedure. Inclusion criteria in the AMI group were typical clinical symptoms, electrocardiographic ST-elevation, and occlusion of a central coronary artery verified by coronary angiography. Inclusion criteria in the AP group were typical clinical symptoms and coronary artery disease angiographically suitable for PCI. Previous transmural infarction, cardiogenic shock, and serious comorbidity were exclusion criteria in both groups. Patients in both groups were treated medically in accordance with current guidelines. Details of the study design have previously been reported [12]. The study protocol was approved by the Regional Committee for Medical Research Ethics. All patients gave informed, written consent.

### 2.2. Blood Sampling and Laboratory Analysis

Blood samples were collected by standard venipuncture immediately before PCI in the AP group and after 3 hours, 12 hours, and 1, 3, 5, 7, and 14 days in both groups. All samples from day 1 and further on were obtained in fasting condition and before intake of any medication. Routine analysis was performed by conventional methods. EDTA-plasma was prepared by centrifugation within 1 hour at 2500 ×g for 20 min at 4°C and stored at −80°C until analysed.

Commercial Enzyme Linked Immunosorbent Assays (ELISA) (R&D Systems, Abingdon, Oxon, UK) were used to determine circulating PTX3 levels. Total RNA was extracted from PAXgene Blood RNA tubes by using the PAXgene Blood RNA Kit (PreAnalytix, Qiagen GmbH, Germany), including an extra cleaning step (Rneasy MinElute Cleanup Kit, Qiagen). A total of 100 ng RNA (range 200–800 ng/*μ*L) was reversely transcribed in a total volume of 20 *μ*L in all samples using the Omniscript RT Kit (Qiagen), Oligos (dTs), and Rnase Inhibitor (Applied Biosystems, Foster City, CA, USA). PTX3 mRNA levels were determined by real-time PCR on the ViiA 7 Real-Time PCR System (Applied Biosystems), including the TaqMan Gene Expression Assay (HS00173615_ml) and TaqMan Fast Universal PCR Master Mix (2X) No AmpErase UNG (Applied Biosystems). The PTX3 mRNA levels were normalized to *β*-2-microglobulin (HS99999907_ml, Applied Biosystems) and fold expression (relative quantification using the ΔΔ Ct method) was determined in relation to a reference sample, as previously described [13].

### 2.3. Markers of Myocardial Injury

Troponin T (reference value < 0.03 *μ*g/L) and creatine kinase MB (reference values < 5 *μ*g/L) were analysed in serum by conventional routine methods.

### 2.4. Magnetic Resonance Imaging

Cardiac magnetic resonance imaging using a 1,5 T whole body scanner (Philips Intera, Best, The Netherlands) was performed after 6 weeks in the AMI group. Left ventricular volume and ejection fraction were calculated on basis of short axis images. Gadolinium late contrast enhancement technique was used to determine infarct size.

### 2.5. Statistical Analyses

Demographic variables are given as proportions or medians (25, 75 quartiles). Nonparametric statistics were used throughout as PTX3 levels were not normally distributed. Medians and 25 and 75 quartiles are given if not otherwise stated. The Mann-Whitney test was used for group comparisons of continuous data. Friedman test was used for analyses of overall change within the groups, and the Wilcoxon test was used for analyses of difference between time points within a group when appropriate. Spearman's rho was calculated for correlation analysis. Level of significance was set to *P* < 0.05. The SPSS software package version 18.0 was used throughout.

## 3. Results

Baseline characteristics of the study populations did not differ between the AMI and AP groups, except from higher rate of previous PCI in the AP group (*P* = 0.008) (Table 1).

### 3.1. Time Profile of Circulating PTX3

**Figure 1:**
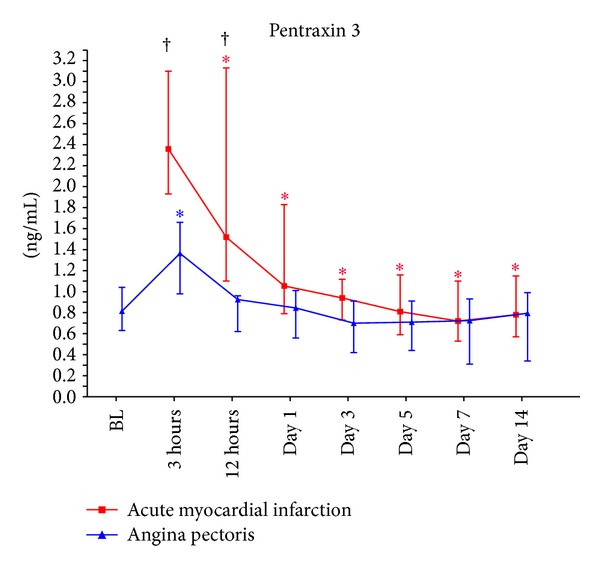
The time profile of circulating PTX3 levels. BL: baseline; ^†^ *P* < 0.05 for between-group differences at the various time points; *P < 0.05 for intragroup differences from baseline in the angina pectoris group and from time point 3 hours in the acute myocardial infraction group.

PTX3 levels at 3 and 12 hours were significantly higher in the AMI group compared to the AP group (2.36 versus 1.37 ng/mL, *P* < 0.001, and 1.52 versus 0.93 ng/mL, *P* = 0.003) (Figure 1).

Within the AMI group, an overall change in circulating PTX3 levels was observed (*P* < 0.001) with significant reduction from 3 to 12 hours, remaining reduced from day 1 throughout the study period (all *P* < 0.001).

Within the AP group, an overall change in circulating PTX3 levels was also observed (*P* < 0.001). In this group there was an increase in the levels from baseline to 3 hours (*P* = 0.022), followed by a reduction from 3 to 12 hours, which sustained reduced throughout (all *P* < 0.05).

### 3.2. Correlations

No significant correlations at different time points between circulating PTX3 levels and degree of myocardial necrosis measured by TnT or infarct size measured by MRI were obtained (Table 2).

Circulating PTX3 levels did not correlate with leukocyte count, except for a negative correlation at day 1 in the AP group (*r* = −0.762; *P* = 0.01).

### 3.3. Genetic Expression of PTX3 in Circulating Leukocytes

The PTX3 mRNA levels were significantly lower in the AMI group compared to the AP group at day 1 (*P* = 0.009). Otherwise no differences between the groups were observed during the study period (data not shown).

**Figure 2:**
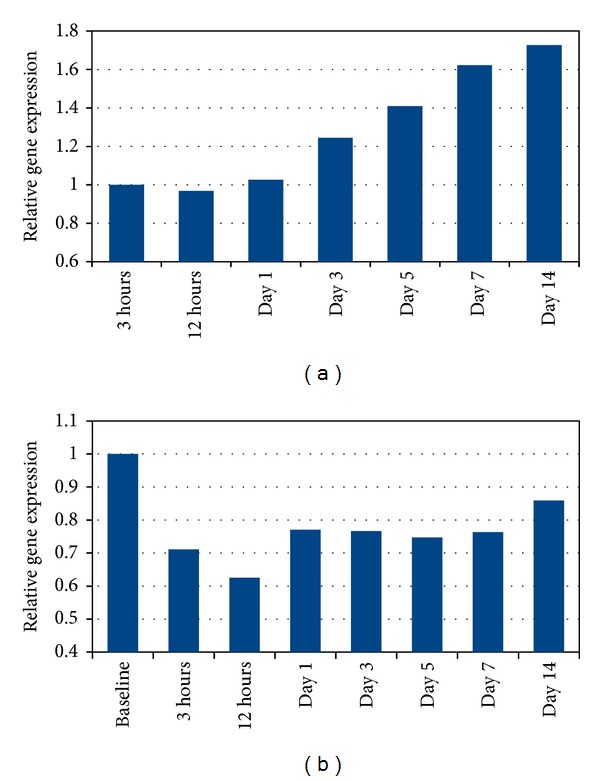
(a) Gene expression of PTX3 in circulating leukocytes relative to 3 hours' levels in the AMI group. (b) Gene expression of PTX3 in circulating leukocytes relative to baseline levels in the AP group.

Within the AMI group, PTX3 mRNA levels increased from 3 hours to days 7 and 14 (*P* = 0.035 and *P* = 0.013, resp.). When defining the 3-hour mRNA values as 1.0, the relative upregulation was 62% and 73%, respectively (Figure 2(a)).

Within the AP group, PTX3 mRNA levels were reduced from baseline to 3 hours, 12 hours, and day 1 (*P* = 0.017, *P* = 0.013, and *P* = 0.037, resp.), followed by sustained reduced levels. When defining baseline mRNA values as 1.0, the relative downregulation at 3 and 12 hours was 29% and 37%, respectively (Figure 2(b)).

No significant correlations were found between circulating PTX3 levels and PTX3 mRNA levels at any time point in either of the groups (data not shown).

## 4. Discussion

The main findings of the study were as follows: (1) circulating PTX3 levels were significantly higher in the AMI group compared to the AP group shortly after PCI, being significantly reduced after 12 hours, (2) circulating PTX3 levels did not correlate to infarct size nor to leukocyte count, and (3) in both groups the genetic expression of PTX3 showed an inverse pattern compared to the circulating levels in a negative feedback manner.

Higher circulating PTX3 levels in AMI patients compared with those in the AP patients short time after PCI indicate that the myocardial infarction per se somehow influences PTX3 levels. As blood samples before PCI were not available in this group, we can only speculate regarding these levels. The possibility that pre-PCI levels would have been even higher might though be indicated. Newly ischemic situations have previously been reported to associate with high PTX3 levels [14]. In line with our results, an early peak of PTX3 levels has been reported in patients with AMI [6]. An immediate source of PTX3 during acute inflammation has been shown to be released from specific granules of neutrophile granulocytes [15]. Once released, PTX3 inhibits both local P-selectin-dependent leukocyte enrollment on endothelial cells and platelet aggregation, presumably in order to dampen the inflammatory process and the subsequent injury of an acute myocardial infarction [4, 8, 9]. These circumstances taken together with reports of increased atherosclerotic burden, increased expression of a diversity of proinflammatory cytokines and adhesion molecules, and increased myocardial damage in studies with PTX3 ^−^/^−^ mice may suggest that the high levels of PTX3 in the AMI group shortly after PCI reflect the anti-inflammatory properties of PTX3 in order to diminish myocardial damage [10, 11]. The subsequent fall in PTX3 levels in the AMI group could be a consequence of emptied stores of PTX3 in circulating leukocytes and other potential PTX3 secreting cells like ischemic cardiomyocytes [16], or that the acute stimuli to secretion, whatever that is, has ceased.

In the AP group, limited but statistically significant changes in PTX3 levels from baseline (before PCI) to 3 hours were observed. We suggest that this rise in circulating PTX3 might be related to the PCI procedure per se. This has to be further explored.

Although obviously influenced by the AMI per se, levels of circulating PTX3 did not correlate neither to size of infarction nor to leukocyte count. As to the first, the mean size of myocardial damage was relatively modest in our AMI group, which may influence the result. Lack of correlation between circulating PTX3 levels and infarct size has, however, also been reported elsewhere [6]. Given the potential role of PTX3 as a prognostic marker of future coronary events and overall mortality, it would have been reasonable to suggest a correlation between PTX3 levels and infarct size. The absence of such a correlation makes it tempting to speculate whether levels of PTX3 predict future adverse events through other mechanisms than the actual size of myocardial necrosis, for instance, through the level of inflammatory burden or response. Another possibility is that neutrophil activation leading to PTX3 release precedes the myocardial infarction or is partially responsible for the plaque rupture [17].

The lack of correlation between circulating PTX3 levels and leukocyte count may indicate that other regulatory mechanisms for PTX3 than the quantitative amount of leukocytes are of importance. It might be that a graded PTX3 release from the granules of neutrophile granulocytes, independent of total leukocyte count, plays a role. Another possibility is release of PTX3 from other cell types than leucocytes, like ischemic cardiomyocytes or activated endothelial cells. The possibility that leucocyte count at 12 h is too late to catch a correlation between quantitative leukocyte count and the immediate secretion of circulating PTX3 during AMI should also be noted.

The genetic expression of PTX3 was upregulated after one week in the AMI group and reduced at 3 hours after PCI in the AP group. The genetic expression profiles of PTX3 seen in both groups might be a compensatory response to the fluctuations seen in the circulating levels in a negative feedback manner. The genetic expression in leukocytes may reflect the amount of circulating PTX3 released by leukocytes. However, PTX3 during an AMI is probably released by both leukocytes and other cells, like activated endothelial cells and injured cardiomyocytes [16]. Therefore, the genetic upregulation observed in circulating leukocytes is probably only partially reflecting the regulatory mechanisms of circulating PTX3.

The small sample size is a limitation of the study. Strength of the study is the experimental model of studying regulatory mechanisms by comparing circulating PTX3 levels to genetic PTX3 mRNA expression in circulating leukocytes. Although knowledge about circulating PTX3 levels during acute myocardial infarctions has increased lately, data on protein levels in relation to the genetic mRNA levels have until now been scarce.

## 5. Conclusion

Elevated circulating PTX3 levels shortly after PCI in AMI patients compared to AP patients indicate that the myocardial infarction per se influences PTX3, although the levels were not correlated to infarct size. The genetic expression of PTX3 in circulating leukocytes showed an inverse pattern, probably compensatory to the fluctuations in the circulating protein levels. The implication of these findings with respect to prognosis in patients with CAD remains to be explored.

## Figures and Tables

**Table 1:** Baseline characteristics of the study populations.

	AMI group (*n* = 20)	AP group (*n* = 10)
Age, years	59.5 (54, 67)	63.5 (54, 71)
Female gender (*n*)	5	1
Hypertension (*n*)	7	4
Diabetes (*n*)	2	2
Smokers (*n*)	7	2
Previous AMI (*n*)	0	2
Previous PCI (*n*)	0	4
Previous ACB (*n*)	0	2
SBP (mmHg)	140 (120, 154)	137 (128, 146)
DBP (mmHg)	85 (81, 98)	85 (75, 100)
TnT max. (ng/L)	3.5 (2.0, 5.7)	
Infarct size (MRI) (%)	6.6 (3.5, 10.7)	
EF (MRI) (%)	57.5 (53.3, 66.3)	
Symptom debut to PCI (min)	145 (95, 410)	
Leukocyte count at 12 h	9.0 (7.8, 10.4)	7.6 (5.6, 8.2)
Medication at discharge		
Aspirin (*n*)	20	10
Clopidogrel (*n*)	20	9
ACE-I/AII antagonist (*n*)	11	2
*β* blocker (*n*)	18	6
Statin (*n*)	20	10

Values are given as proportions or medians (25, 75 percentiles).

ACE-I: angiotensin-converting enzyme inhibitor. AII antagonist: angiotensin II receptor antagonist. *β* blocker: beta adrenergic receptor blocker. ACB: coronary bypass surgery.

DBP: diastolic blood pressure. EF: ejection fraction. PCI: percutaneous coronary intervention. SBP: systolic blood pressure. TnT: Troponin T.

**Table 2:** Correlations between circulating PTX3 levels and infarct size.

	TnT max.		MRI	
	Correlation coefficient^1^	*P* value	Correlation coefficient	*P* value
PTX3				
3 h	−0.364	0.137	0.102	0.718
12 h	−0.158	0.519	−0.031	0.910
Day 1	0.093	0.722	−0.116	0.680
Day 3	−0.138	0.586	−0.339	0.200
Day 5	0.238	0.324	0.179	0.507
Day 7	−0.081	0.751	−0.002	0.994
Day 14	−0.237	0.344	−0.223	0.406

^1^Spearman's rho.

MRI: magnetic resonance imaging; TnT: Troponin T.
